# Diverse Genotypes and Species of *Cryptosporidium* in Wild Rodent Species from the West Coast of the USA and Implications for Raw Produce Safety and Microbial Water Quality

**DOI:** 10.3390/microorganisms9040867

**Published:** 2021-04-17

**Authors:** Xunde Li, Edward Robert Atwill

**Affiliations:** 1 Western Institute for Food Safety and Security, University of California, Davis, CA 95616, USA; xdli@ucdavis.edu; 2 Department of Population Health and Reproduction, School of Veterinary Medicine, University of California, Davis, CA 95616, USA

**Keywords:** *Cryptosporidium*, species, genotypes, wildlife, rodent, zoonotic

## Abstract

*Cryptosporidium* spp. are protozoan parasites that infect perhaps all vertebrate animals, with a subset of species and genotypes that function as food- and waterborne pathogens. The objective of this work was to collate the *Cryptosporidium* species and genotypes from common wild rodents on the west coast of the USA and update the information regarding the zoonotic potential of *Cryptosporidium* from these ubiquitous wild species. Representative sequences of the 18S rRNA gene for a unique set of *Cryptosporidium* isolates obtained from deer mice, house mice, mountain beavers, yellow-bellied marmot, long-tailed vole, California ground squirrels, Belding’s ground squirrels, and a golden-mantled ground squirrel in GenBank were selected for phylogenetic analysis. Phylogenetic and BLAST analysis indicated that 4 (18%) of the 22 unique *Cryptosporidium* sequences from these wild rodent species were 99.75% to 100% identical to known zoonotic species (*C. parvum*, *C. ubiquitum*, *C. xiaoi*), suggesting that a minority of these representative *Cryptosporidium* isolates could have a public health impact through food and waterborne routes of human exposure. These zoonotic isolates were shed by deer mice and a yellow-bellied marmot from California, and from a mountain beaver trapped in Oregon. In addition, the group of unique *Cryptosporidium* isolates from deer mice and ground dwelling squirrels exhibited considerable DNA diversity, with multiple isolates appearing to be either host-limited or distributed throughout the various clades within the phylogenetic tree representing the various *Cryptosporidium* species from host mammals. These results indicate that only a subset of the unique *Cryptosporidium* genotypes and species obtained from wild rodents on the US west coast are of public health concern; nevertheless, given the geographic ubiquity of many of these host species and often high density at critical locations like municipal watersheds or produce production fields, prudent pest control practices are warranted to minimize the risks of water- and foodborne transmission to humans.

## 1. Introduction

*Cryptosporidium* spp. infect a wide range of vertebrate animals, including humans [1,2]. During the past two decades, new species have been continuously discovered for the *Cryptosporidium* genus. Among the nearly 40 species that have been described, 3 species infect fish (*C. huwi*, *C. molnari*, *C. scophthalmi*); 1 species infects amphibians (*C. fragile*); 4 species infect reptiles (*C. ducismarci*, *C. serpentis*, *C. testudinis*, *C. varanii*); 5 species infect birds (*C. avium*, *C. baileyi*, *C. galli*, *C. meleagridis*, *C. proventriculi*); and 24 species infect various species of mammals (*C. andersoni*, *C. apodemi*, *C. bovis*, *C. canis*, *C. cuniculus*, *C. ditrichi*, *C. erinacei*, *C. fayeri*, *C. felis*, *C. hominis*, *C. macropodum*, *C. muris*, *C. occultus*, *C. parvum*, *C. proliferans*, *C. rubeyi*, *C. ryanae*, *C. scrofarum*, *C. suis*, *C. tyzzeri*, *C. ubiquitum*, *C. viatorum*, *C. wrairi*, *C. xiaoi*) [3,4,5,6,7,8,9,10].

According to reviews regarding *Cryptosporidium* spp. that infect humans and therefore represent a risk to public health [4,10], *Cryptosporidium* spp. that are considered zoonotic include (major vertebrate hosts in parenthesis): *C. andersoni* (cattle), *C. bovis* (cattle), *C. canis* (dogs), *C. cuniculus* (rabbits), *C. erinacei* (tree squirrels), *C. fayeri* (kangaroo), *C. felis* (cats), *C. meleagridis* (turkeys), *C. muris* (mice), *C. parvum* (cattle), *C. scrofarum* (pigs), *C. suis* (pigs), *C. tyzzeri* (mice), *C. ubiquitum* (cattle), and *C. xiaoi* (sheep and goats). In addition, *Cryptosporidium* sp. chipmunk genotype I (chipmunks), horse genotype (horses), mink genotype (minks), and skunk genotype (skunks) have also been associated with human infections [4]. Among these zoonotic species and genotypes, *C. hominis* and *C. parvum* are responsible for the majority of human infections [10,11] and the majority of waterborne outbreaks in human communities [5].

Rodents represent the largest order of living mammals [12]. North America has diverse and in some cases distinctive rodent species, such as the most primitive of all living rodents (mountain beavers of the family Aplodontidae), the diverse family of Sciuridae (squirrels), the ubiquitous and large family of Cricetidae (mice and rats), and some globally-common rodents of the large family Muridae [13]. Rodents are widespread in diverse habitats and are often the most abundant mammals in those habitats. For example, deer mice (*Peromyscus maniculatus*) are abundant and widely distributed rodents in North America that occupy diverse habitats, including watersheds, urban centers, and agricultural landscapes such as produce fields in central coastal California [14,15]. Rodents are recognized as host reservoirs of more than 60 zoonotic infectious agents that, as a consequence, are threats to public health [16,17]. Rodents can transmit pathogens to humans in a variety of mechanisms: manual handling or direct contact with rodents; physical contact with rodent feces, urine, or saliva; through rodent bites [16,18]; or indirectly when rodents contaminate raw foods or drinking water supplies with zoonotic pathogens that are subsequently ingested by humans [15,19,20]. For example, rodents are reservoirs or can transmit salmonellosis, plague, leptospirosis, and rat-bite fever [21]. The aim of this work was to consolidate the diversity of *Cryptosporidium* spp. and genotypes shed in the feces of wild rodents that we and other researchers have documented over the past decade of research [15,20,22,23,24,25,26,27] and to update the phylogenetic relationships of unique *Cryptosporidium* sequences from wild rodents on the west coast of the United States (US); this information will reveal new public health implications for food safety and water quality for the different species and genotypes of *Cryptosporidium* in wild rodents from this geographical region of the US.

## 2. Materials and Methods

### 2.1. Selection of Cryptosporidium Sequences of Wild Rodents from GenBank

Based on the literature of *Cryptosporidium* spp. in wild rodent hosts trapped from the west coast of the US [15,20,22,23,24,25,26,27,28] and through online queries for DNA sequences of *Cryptosporidium* from this region of the US in the publicly accessible GenBank (https://www.ncbi.nlm.nih.gov/genbank/, accessed on 28 December 2020), 36 total isolates were identified, resulting in 22 unique sequences of the 18S rRNA gene of *Cryptosporidium*. These DNA sequences of *Cryptosporidium* isolates were from multiple deer mice (*Peromyscus maniculatus*), a house mouse (*Mus musculus*), mountain beavers (*Aplodontia rufa*), yellow-bellied marmots (*Marmota flaviventris*), a long-tailed vole (*Microtus longicaudus*), multiple California ground squirrels (*Otospermophilus beecheyi*), Belding’s ground squirrels (*Urocitellus beldingi*), and a golden-mantled ground squirrel (*Callospermophilus lateralis*). In several cases, there were multiple isolates of *Cryptosporidium* with 100% sequence similarity for a specific host species (n = 14, total); in those cases, only a single representative sequence was selected for this analysis (i.e., only non-redundant sequences were included) which reduced the total number of DNA sequences from 36 isolates to 22 unique (non-redundant) sequences, as explained in detail in the next paragraph.

**Figure 1:**
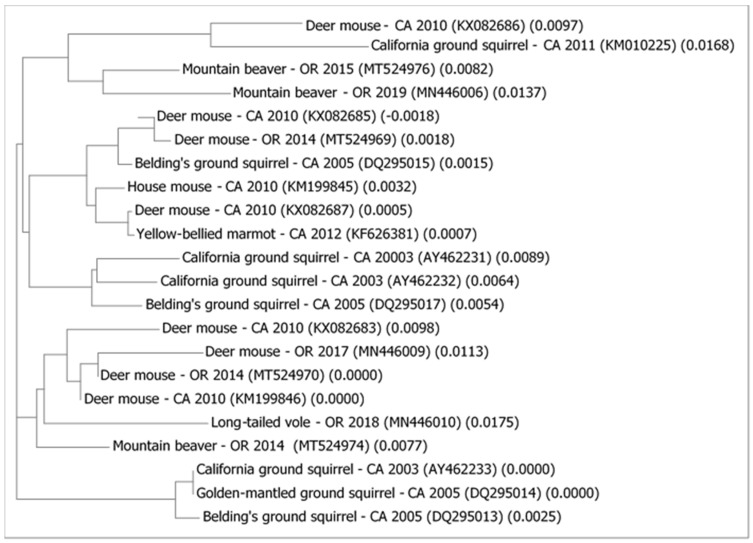
Phylogenetic relationships among relatively recent *Cryptosporidium* isolates from wild rodent species from the west coast of the US (California (CA), Oregon (OR)). GenBank accession numbers in the brackets after the name of the host species of rodent, its geographical location, and year when the isolate was obtained.

There was only one DNA sequence each available from a long-tailed vole in Oregon, a golden-mantled ground squirrel in California, and a house mouse in California; hence, all these sequences were selected for our analysis. The DNA query found 7 sequences from deer mice from California, 10 sequences from deer mice from Oregon, 2 sequences from yellow-belied marmots from California, 5 sequences from mountain beavers from Oregon, 6 sequences from California ground squirrels from California, and 3 sequences from Belding’s ground squirrels from California. In these cases, sequences from the same host species and location were preliminary analyzed using the NCBI BLAST’s “Align two or more sequences” function and only sequences <99.5% homogeneity were selected for this work. As a result, 5 sequences from deer mice in California, 3 sequences from deer mice in Oregon, 4 sequences from California ground squirrels in California, 3 sequences from Belding’s ground squirrels in California, 3 sequences from mountain beavers in Oregon, and 1 sequence from a yellow-bellied marmot in California were selected. The process used for the selection of *Cryptosporidium* sequences for this analysis is shown in Figure S1. GenBank accession numbers of selected *Cryptosporidium* sequences from rodents and locations of samples collected from host species are shown in Figure 1 and Table 1.

### 2.2. Phylogenetic Analysis

**Figure 2:**
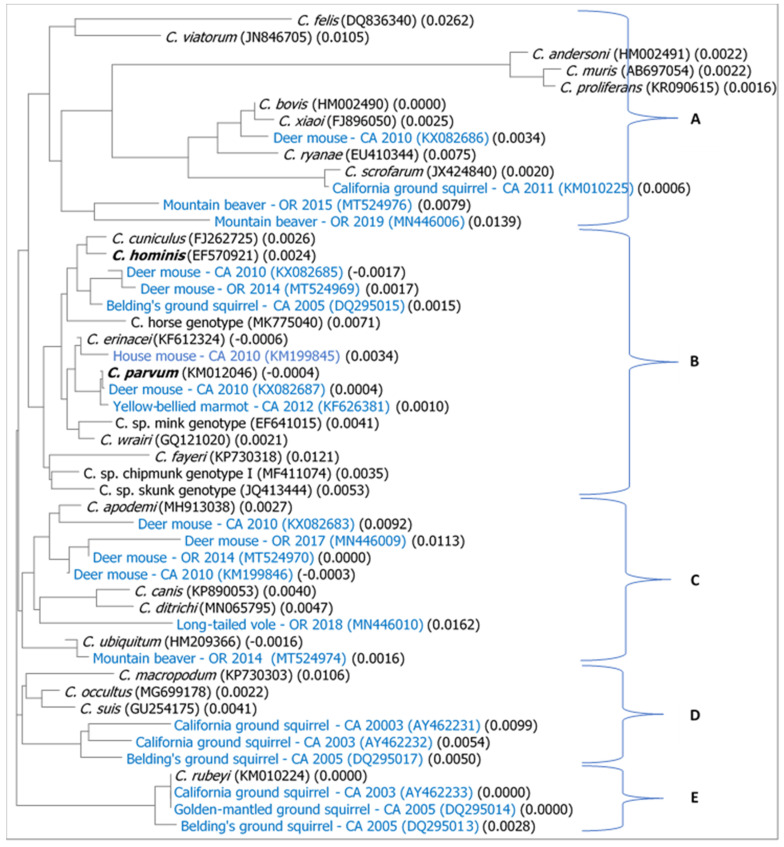
Phylogenetic relationship between relatively recent *Cryptosporidium* isolates from wild rodent species from the west coast of the US (**blue font**) and a collection of representative *Cryptosporidium* species from mammals and selected zoonotic genotypes (**black font**). Given their importance in human infection, *C. homonis* and *C. parvum* are in bold.

Two phylogenetic analyses were conducted: first, the phylogenetic relationships among all selected *Cryptosporidium* spp. from wild rodents trapped on the west coast of the US; second, the phylogenetic relationships between *Cryptosporidium* from wild rodents and named reference *Cryptosporidium* species in mammals (including humans) and known reference zoonotic genotypes. Depending on the availability of the 18S rRNA gene sequences of *Cryptosporidium* in the GenBank, reference sequences for the second phylogenetic analysis were selected as previously described [27] and based on: (1) sequences of isolates representing described *Cryptosporidium* species from mammals; (2) sequences of isolates from known zoonotic genotypes; (3) sequences previously used by other investigators for species description or as reference sequences; (4) sequence length (longer sequence available for each species; i.e., ≥700 bp); and (5) sequences not originating from cloned PCR products due to the potential for erroneous sequence data generated from cloning PCR products [29,30]. Phylogenetic trees were constructed using the Vector NTI Advance 11 based on a pairwise alignment. GenBank accession numbers of the selected reference sequences are available in Figure 2.

### 2.3. BLAST Analysis

To compare our bank of wild rodents *Cryptosporidium* isolates with existing reference species and genotypes of *Cryptosporidium* in the GenBank, every selected wild rodent *Cryptosporidium* sequence was aligned with other *Cryptosporidium* sequences in the GenBank using the NCBI’s online nucleotide basic local alignment search tool (BLAST). The BLAST analysis was optimized for highly similar sequences using default algorithm parameters and 100 maximum target sequences (31 December 2020 as last day accessed).

The rationale for conducting this BLAST analysis was that comparative genotyping is commonly used to broadly characterize the zoonotic or human-infection risk for a novel isolate of *Cryptosporidium*. For example, if the DNA sequence for a reasonably long section of the 18S rRNA gene from a *Cryptosporidium* isolate is either highly related (≥99.5%) or has 100% sequence homogeneity to a known zoonotic species or genotype, the isolate is typically considered to be infectious to humans. In contrast, if the DNA sequence for an isolate is not highly related to any known zoonotic species or genotypes of this parasite, it is generally considered not infectious to humans. Although this decision process is not perfect, it is a current convention used by many US state and federal regulatory agencies to assign zoonotic disease risk to an isolate of *Cryptosporidium* found either in water or on food.

## 3. Results

### 3.1. Diverse Genotypes of Relatively Recent Cryptosporidium Isolates from Wild Rodents throughout the West Coast, US

Phylogenetic relationships among the 22 unique (non-redundant) 18S rRNA gene sequences from 36 isolates of *Cryptosporidium* from wild rodents trapped from the US west coast are shown in Figure 1. *Cryptosporidium* isolates from deer mice were widely distributed among various clades demonstrating considerable genomic diversity within this host species: two deer mouse isolates from California (KX082683 and KM199846) and two deer mouse isolates from Oregon (MN446009 and MT524970) formed one clade; a fifth deer mice isolate from California (KX082685) and a sixth deer mouse isolate from Oregon (MT524969) were located in the second clade containing a closely related *Cryptosporidium* isolate from a Belding’s ground squirrel from California, along with isolates from a California house mouse, *C. parvum* isolate from a California yellow-bellied marmot, and an isolate that was 99.88% similar to *C. parvum* from the seventh deer mouse isolate (KX082687) from California. The last deer mouse isolate (KX082686) from California was distinct from all other deer mouse isolates and was located in a clade of relatively unrelated and distinct isolates from a California ground squirrel (KM010225) and two mountain beaver isolates (MT524976 and MN446006) from Oregon. The third mountain beaver strain (MT524974) was distinct from these first two mountain beaver strains and formed a clade with relatively unrelated *Cryptosporidium* isolates from four deer mice discussed above and a long-tailed vole (MN446010) from Oregon. In contrast to the deer mouse isolates that were distributed throughout various clades, the majority of isolates from the three species of ground squirrels formed their own separate clades, with the first clade containing two isolates from California ground squirrels (AY462231, AY462232) and an isolate from a Belding’s ground squirrel (DQ295017), while the second clade contained one isolate each from three species of ground squirrels: California ground squirrel (AY462233), golden-mantled ground squirrel (DQ295014), and a Belding’s ground squirrel (DQ295013).

### 3.2. Phylogenetic Relationships between Relatively Recent Cryptosporidium Isolates from Wild Rodents from the West Coast and Representative Cryptosporidium Species and Zoonotic Genotypes

The phylogenetic relationships between *Cryptosporidium* from west coast wild rodents and selected representative *Cryptosporidium* species from mammals and known zoonotic genotypes are shown in Figure 2. The phylogenetic tree for this collection of known *Cryptosporidium* species, genotypes, and west coast rodent isolates is comprised of roughly five major clades (A, B, C, D, and E), with the majority of west coast rodent isolates distributed widely across the five major clades of mammalian-derived *Cryptosporidium*. Clade A is composed of four minor but distinct sub-clades, including one containing the three distinct species of gastric *Cryptosporidium* found in mammals (Figure 2) and a separate sub-clade containing *C. felis* (DQ836340) and *C. viatorum* (JN846705). In addition, the two mountain beaver isolates (MT524976 and MN446006) form a distinct sub-clade within this larger clade A of relatively unrelated isolates. Clade B contains the two most common causes of human infection from this protozoal parasite, *C. hominis* and *C. parvum*, each in a different sub-clade, along with a large group of known *Cryptosporidium* species and genotypes and highly related isolates of rodent *Cryptosporidium* from the west coast based on DNA sequence similarity. Clade C is dominated by isolates of rodent *Cryptosporidium*: deer mouse isolates from California (KX082683 and KM199846) and Oregon (MN446009 and MT524970), an isolate from a long-tailed vole from Oregon (MN446010), an isolate from a mountain beaver from Oregon (MT524974), and known species such as *C. ubiquitum* (HM209336), *C. canis* (KP890053), and *C. ditrichi* (MN065795). The smaller clade D contains a sub-clade of highly related isolates from ground-dwelling squirrels from California (California and Belding’s ground squirrels) and a subclade of three known species, *C. macropodum* (KP730303), *C. occultus* (MG699178), and *C. suis* (GU254175). Lastly, clade E is comprised entirely of highly related *Cryptosporidium* isolates from three major species of ground-dwelling squirrels in California (California, Belding’s, golden-mantled ground squirrels) and a relatively newly described species of *Cryptosporidium*, *C. rubeyi* (KM010224) found in all three of these species of squirrel.

### 3.3. Comparison of Cryptosporidium from Wild Rodents with Cryptosporidium Species and Genotypes Deposited in GenBank

The comparison of the collection of wild rodent isolates of *Cryptosporidium* from the west coast of the US to known *Cryptosporidium* species and genotypes in the GenBank is shown in Table 1. The majority of deer mouse strains from California and Oregon are 99.15–100% identical to various prior isolates from deer mice, with the exception that one deer mouse isolate from California (KX082687) was nearly identical (99.88%) to *C. parvum* (MT071829) and a second deer mouse isolate from California was also nearly identical (99.75%) to *C. xiaoi* (MH049731). Complete homogeneity (100% identical) was found between a California house mouse isolate (KM199845) and a prior mouse genotype (EU553589) and between the yellow-bellied marmot isolate from California (KF626381) and *C. parvum* (KU892559). The isolate from a long-tailed vole strain (MN446010) was 99.73% similar to an isolate from environmental water (KF994580) [31]. Among the three mountain beaver isolates from Oregon, one (MT524974) was 100% identical to a *C. ubiquitum* isolate (KC608024), another (MT524976) was 98.02% similar to an isolate from a bobcat (MT524975) from the same Oregon location, and the last isolate (MN446006) was relatively unique and only 97.95% similar to the reptilian *C. ducismarci* (MF737079), suggesting this is a new genotype or cryptic species of *Cryptosporidium*. The California ground squirrel strain (AY462233) was 100% identical to Sbey05c (DQ295012; not shown in Table 1) and Sbey11c (KM010224) that we detected from the same squirrel species across different years and was eventually named as a new species, *C. rubeyi* [25]. The California ground squirrel strain (KM010225) was 99.75% similar to *C. scrofarum* (MH178036). The Belding’s ground squirrel strain (DQ295015) was 99.76% similar to an isolate from a deer mouse in California (KM199844). Maximum percent identities for all other strains from the three ground squirrel species were 97.85–99.83% similar to themselves and therefore not highly related to any of the currently known mammalian strains of *Cryptosporidium*. For example, both a 2003 isolate (AY462232) from a California ground squirrel and a 2005 isolate (DQ295017) from a Belding’s ground squirrel were only 97.85% similar to previous isolates of ground squirrels, indicating that despite the large number of newly described *Cryptosporidium* species since 2003–2005, these unique strains of *Cryptosporidium* remain unrelated (i.e., poor sequence homogeneity) to any known mammalian species or genotypes of *Cryptosporidium* and likely indicate a new genotype or cryptic species of this protozoal parasite.

## 4. Discussion

### 4.1. The Selection of Representative Sequences of Cryptosporidium from Rodents

Given that the 18S rRNA gene is commonly used for speciation and/or molecular characterization when comparing new isolates against known species and genotypes of *Cryptosporidium* [11], we compared the 18S rRNA gene sequences in GenBank for isolates of *Cryptosporidium* from rodent species from the west coast of the US to known *Cryptosporidium* species and genotypes isolated from mammals for this comparative analysis. Only one sequence had been submitted, or only one unique sequence was available in GenBank, for isolates of *Cryptosporidium* from several rodent species in this phylogenetic analysis of *Cryptosporidium* isolates from rodents from the west coast of the US; for example, a single sequence of *Cryptosporidium* was available for long-tailed voles, golden-mantled ground squirrels, and yellow-bellied marmots. In contrast, there were ≥3 unique and available sequences for our comparative analysis of deer mice in California, deer mice in Oregon, mountain beavers in Oregon, and California ground squirrels and Belding’s ground squirrels from California. No *Cryptosporidium* 18S rRNA gene sequences from rodent host species were found from the state of Washington in the GenBank.

### 4.2. Diversity of Cryptosporidium in Wild Rodents on the West Coast, US

Phylogenetic and BLAST analysis indicated a diversity of *Cryptosporidium* species and genotypes circulating in these wild rodent species. Interestingly, sequence homogeneity with known *Cryptosporidium* species, along with isolates exhibiting sequence heterogeneity, were observed among the collection of isolates from deer mice from California and Oregon. For example, *Cryptosporidium* sequences from a deer mouse in California (KX082685) and a deer mouse in Oregon (MT524969) were in clade B of Figure 2, which contains a large diversity of known *Cryptosporidium* species and genotypes, while *Cryptosporidium* sequences from a different group of deer mice in California (KX082683, KM199846) and in Oregon (MT524970, MN446009) were located in a completely different clade C (Figure 2). In contrast, sequences from two deer mice in California (KX082686, KX082687) were not closely related to other rodent isolates; instead, they were closely related (≥99.75% sequence similarity) to either *C. parvum* or *C. xiaoi*. Among the three different sequences isolated from mountain beavers from a watershed in northwestern Oregon, two formed a unique subclade within clade A and the other mountain beaver isolate grouped with deer mice and other species of *Cryptosporidium* (Figure 1 and Figure 2), indicating that multiple genotypes of *Cryptosporidium* from mountain beavers can occur for these hosts even when trapped from the same watershed location. For sequences from California ground squirrels and Belding’s ground squirrels, results were consistent with previous reports that these genotypes mainly infect and circulate within the common three species of ground squirrels [23] that were eventually named as a new species, *C. rubeyi*, based on the c-genotype [25]. Exceptions were that the Sbey11e (KM010225) from California ground squirrels and the Sbld05d (DQ295015) from Belding’s ground squirrels are more closely related to isolates from deer mice.

Eighteen percent (4/22) of these non-redundant *Cryptosporidium* sequences from various rodent host species from the US west coast were 99.75–100% homogeneous to known species of zoonotic *Cryptosporidium* (*C. parvum*, *C. ubiquitum*, *C. xiaoi*). Despite this relatively low proportion of non-redundant sequences being highly related to zoonotic rodent C*ryptosporidium* among the total set of 22 unique sequences of rodent *Cryptosporidium*, caution is warranted over the interpretation of this 18% value. Prior studies have shown, for example, that almost 50% of the *Cryptosporidium* isolates obtained from deer mice in California agricultural fields were 99.88 to 100% homologous to *C. parvum* strains in the GenBank, with the majority of these zoonotic isolates being 100% homologous to each other. Hence, in this current analysis we only selected one representative sequence from this group of highly related strains, so the 22 unique sequences of rodent *Cryptosporidium* are un-weighted with respect to the proportion of zoonotic *Cryptosporidium* isolates being shed in the feces of a population of wild rodents. Nevertheless, the lower sample size suggests that this finding should be interpreted with caution and more sequences from a broader geographical region would be warranted for future phylogenetic analysis.

### 4.3. Water Quality and Produce Safety Implications from Zoonotic Cryptosporidium in Rodents

*Cryptosporidium* is a common pathogen that causes enteric disease via waterborne outbreaks [4]. The largest documented waterborne outbreak of cryptosporidiosis occurred in Milwaukee, WI, USA in 1993, which was transmitted through the public water supply to infect over 400,000 people [32]. A community outbreak of cryptosporidiosis associated with surface water-supplied municipal water also occurred on the US west coast (Baker City, Oregon) in 2013, in which 2780 people were infected [33]. *Cryptosporidium* spp. oocysts have been detected in numerous municipal and rural watersheds; for example, in Canada [34,35], Luxembourg [36], and France [37]. In the US, detection of *Cryptosporidium* spp. oocysts in watersheds has been reported on the east [38,39,40] and west coast (California [41], Washington [42], Oregon [27]). Given that *Cryptosporidium* in wild rodents such as deer mice, yellow-bellied marmots, and mountain beavers can be >99.75% homologous to *C. parvum*, *C. ubiquitum,* or other zoonotic species or genotypes of *Cryptosporidium*, along with the observation that rodents like deer mice can shed very high concentrations (>10^8^) of oocysts per g feces [24], reasonable control measures may be needed to prevent high population densities of rodents occurring in close proximity to recreational water sources or municipal drinking water supplies that can pose a risk for waterborne transmission to humans and other animals.

Similar to the above public health concerns regarding the risk of waterborne contamination of critical water supplies, there is growing concern regarding the risk of zoonotic *Cryptosporidium* contamination of human produce consumed raw or with minimal processing. For example, apple juice has been associated with several cryptosporidiosis outbreaks [43,44,45]. *Cryptosporidium* contamination of fresh vegetables or fruits has been widely reported in many countries, including Brazil [46], China [47], Costa Rica [48], Ghana [49,50], India [51], Iran [52], Italy [53], Korea [54,55], Norway [56], Peru [57], Poland [58], and Spain [59]. Two large foodborne outbreaks of confirmed cryptosporidiosis were associated with the consumption of fresh salad, with one outbreak in the UK associated with the consumption of fresh pre-cut salad leaves in which over 300 cases were involved [60] and the other outbreak in Finland associated with Frisée salad, in which 250 individuals became ill [61]. Given the proximity of many fruits and vegetables that are grown on beds close to the soil surface (e.g., strawberries, cantaloupe, lettuce, spinach, cilantro), these commodities are at risk of microbial contamination from rodent feces leading to fecal splash or contaminated furrow water during overhead or foliar irrigation [62]. Rodent populations that forage or establish natal burrows near and within produce fields can result in high levels of protozoal contamination of the soil surface in the produce production environment [20,63]. For example, based on fecal testing and mark-recapture to generate rodent density estimates in central coastal California farms, there was an average of 21 deer mice per ha that collectively shed ~5 × 10^8^ oocysts per day into the environment [20]. Deer mice in this geographical region were infected with *Cryptosporidium* that was highly DNA sequence similar to *C. parvum* (99.88–100%) [20], with *C. parvum* oocysts in fecal material capable of extensive environmental survival during the cooler months of the year [64]. Lastly, rodent predation of produce commodities like strawberries that are grown on the ground can result in elevated pathogen contamination; for example, in Guangzhou, China, there was a higher risk of *E. coli* and *Cryptosporidium* contamination on strawberries that had evidence of rodent damage (bitten) compared to strawberries without any evidence of rodent damage [19], likely due to rodents inadvertently contaminating the surface of strawberries with oocysts when they manually handled and bit the strawberries.

Despite the relatively small body mass of individual rodents compared to livestock and other larger mammalian wildlife species, the risks of food- and waterborne transmission of zoonotic *Cryptosporidium* are elevated by the large number of diverse species in the order Rodentia, their ubiquitous geographical distribution from high elevation-montane to lowland-coastal valleys, their ability to form dense populations and focal colonies on municipal watersheds and produce farms when conditions favor reproduction, and the documented occurrence of zoonotic species and genotypes *Cryptosporidium* in these host species as updated in this current work.

## 5. Conclusions

The current work focused on the genomic diversity of *Cryptosporidium* in wild rodents from the west coast of the US and to quantify the proportion of unique isolates exhibiting zoonotic infection risk from contaminated produce and water. Based on the group of available sequences of *Cryptosporidium* in the GenBank from wild rodents from throughout the west coast of the US, a minority of these *Cryptosporidium* isolates were highly related to known zoonotic species or genotypes of *Cryptosporidium*. Although this suggests limited public health impact from produce or waterborne contamination, infected rodents can develop highly concentrated populations in close proximity to produce fields and drinking water supplies, which elevates their ability to contaminate the environment with zoonotic Cr*yptosporidium*. Future work is warranted to expand this phylogenetic analysis of *Cryptosporidium* in wild rodents to other geographical locations of the world in order to generate deeper insights regarding the potential public health impacts of *Cryptosporidium* infection from wild rodent populations.

## Figures and Tables

**Table 1:** Comparison of relatively recent isolates of *Cryptosporidium* sp. from wild rodents to 18S rRNA genes of *Cryptosporidium* spp., genotypes, and isolates in GenBank.

*Cryptosporidium* in Rodents in the West Coast (California, Oregon)			Highly Similar Sequences in GenBank by BLAST Analysis (Last Access at 31 December 2020)		
Rodent Host Species	Location/Year	GenBank Accession No.	*Cryptosporidium* Species and Isolates	GenBank Accession No.	Max. Identity (%)
Deer mouse (*Peromyscus maniculatus*)	CA, 2010	KX082687	*Cryptosporidium parvum* isolate 438–723	MT071829	99.88
Deer mouse (*Peromyscus maniculatus*)	CA, 2010	KX082686	*Cryptosporidium xiaoi* isolate AH S1	MH049731	99.75
Deer mouse (*Peromyscus maniculatus*)	CA, 2010	KX082685	*Cryptosporidium* sp. isolate Deer mouse 2014_PNWR6C	MT524969	100
Deer mouse (*Peromyscus maniculatus*)	CA, 2010	KX082683	*Cryptosporidium* sp. isolate 1848-Pero-NA	KY644646	99.15
Deer mouse (*Peromyscus maniculatus*)	CA, 2010	KM199846	*Cryptosporidium* sp. deer mouse genotype IV (W3) isolate CRY1811	JQ413348	99.75
Deer mouse (*Peromyscus maniculatus*)	OR, 2014	MT524969	*Cryptosporidium* sp. ex *Peromyscus maniculatus* isolate 2951	KX082685	100
Deer mouse (*Peromyscus maniculatus*)	OR, 2017	MN446009	*Cryptosporidium* sp. isolate Deer mouse 2014_PNW1052	MT524970	98.87
Deer mouse (*Peromyscus maniculatus*)	OR, 2014	MT524970	*Cryptosporidium* sp. deer mouse genotype IV (W3) isolate CRY1811	JQ413348	99.75
House mouse (*Mus musculus*)	CA, 2010	KM199845	*Cryptosporidium* mouse genotype isolate J46	EU553589	100
Yellow-bellied marmot (*Marmota flaviventris*)	CA, 2012	KF626381	*Cryptosporidium parvum* isolate Swec402	KU892559	100
Long-tailed vole (*Microtus longicaudus*)	OR, 2018	MN446010	*Cryptosporidium* sp. C5605-1st	KF994580	99.73
Mountain beaver (*Aplodontia rufa*)	OR, 2014	MT524974	*Cryptosporidium ubiquitum* isolate A2	KC608024	100
Mountain beaver (*Aplodontia rufa*)	OR, 2015	MT524976	*Cryptosporidium* sp. isolate Bobcat 2014_PNW1108	MT524975	98.02
Mountain beaver (*Aplodontia rufa*)	OR, 2019	MN446006	*Cryptosporidium ducismarci* strain R17-999	MF737079	97.95
California ground squirrel (*Otospermophilus beecheyi*)	CA, 2003	AY462233	*Cryptosporidium rubeyi*	KM010224	100
California ground squirrel (*Otospermophilus beecheyi*)	CA, 2003	AY462231	*Cryptosporidium* sp. Sbld05a	DQ295017	98.60
California ground squirrel (*Otospermophilus beecheyi*)	CA, 2003	AY462232	*Cryptosporidium* sp. Sbld05a	DQ295017	97.85
California ground squirrel (*Otospermophilus beecheyi*)	CA, 2011	KM010225	*Cryptosporidium scrofarum* isolate Henan KB	MH178036	99.75
Belding’s ground squirrel (*Urocitellus beldingi*)	CA, 2005	DQ295015	*Cryptosporidium* sp. wild rodent strain isolate 1134 (deer mouse)	KM199844	99.76
Belding’s ground squirrel (*Urocitellus beldingi*)	CA, 2005	DQ295017	*Cryptosporidium* sp. Sbey03b	AY462232	97.85
Belding’s ground squirrel (*Urocitellus beldingi*)	CA, 2005	DQ295013	*Cryptosporidium* sp. Sltl05c	DQ295014	98.98
Golden-mantled ground squirrel (*Callospermophilus lateralis*)	CA, 2005	DQ295014	*Cryptosporidium* sp. Sbey05c	DQ295012	99.83

## Data Availability

The DNA sequences of *Cryptosporidium* from rodent host species on the west coast, USA used in this study are available at https://www.ncbi.nlm.nih.gov/nuccore (accessed on 28 December 2020), with accession number of each sequences cited in the text of the article.

## Supplementary Materials

The following are available online at https://www.mdpi.com/article/10.3390/microorganisms9040867/s1, Figure S1: Schematic flow chart for selection of *Cryptosporidium* 18S rRNA sequences obtained from wild rodents trapped from the West Coast, USA.
