# Supplementary material for: Diverse Genotypes and Species of Cryptosporidium in Wild Rodent Species from the West Coast of the USA and Implications for Raw Produce Safety and Microbial Water Quality
Source: Microorganisms. 2021 Apr 17;9(4):867. doi: 10.3390/microorganisms9040867 (PMC8073747; doi:10.3390/microorganisms9040867)
Supplement: Supplementary file 1 [file microorganisms-09-00867-s001.zip › microorganisms-1169467-supplementary.pdf]

microorganisms

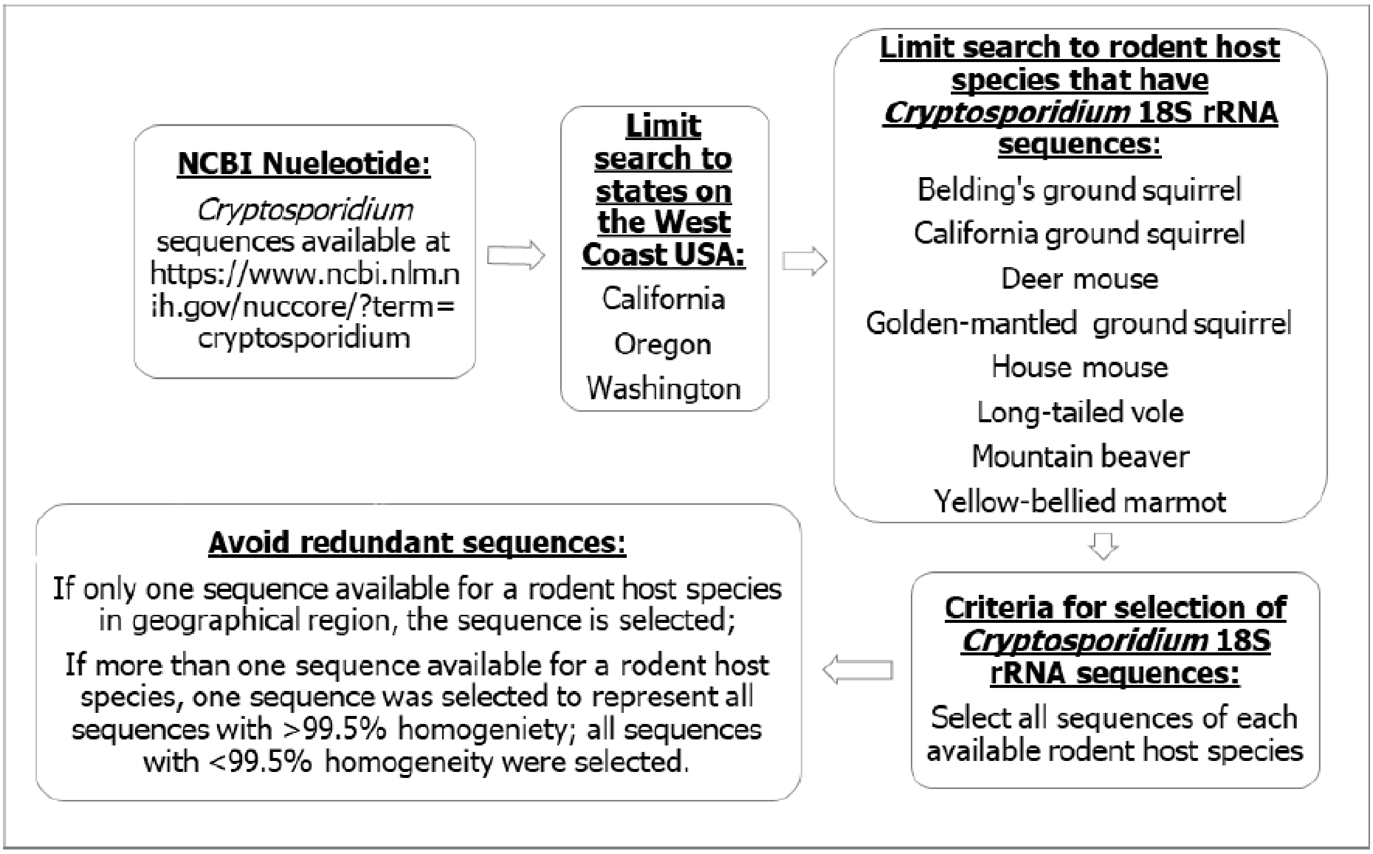

Figure S1. Schematic flow chart for selection of *Cryptosporidium* 18S rRNA sequences obtained from wild rodents trapped from the west coast, USA.
